# Structural and Hormonal Changes Associated With Starvation in Zambian Adult Patients With Esophageal Strictures: A Cross‐Sectional Study

**DOI:** 10.1002/hsr2.72772

**Published:** 2026-07-11

**Authors:** Ellen Besa, Violet Kayamba, Victor Mudenda, Chola Mulenga, Enala Musheba, Malambo Mubbunu, Gary Frost, Paul Kelly

**Affiliations:** ^1^ Tropical Gastroenterology & Nutrition Group University of Zambia School of Medicine Lusaka Zambia; ^2^ Division of Digestive Diseases, Department of Metabolism, Digestion and Reproduction, Faculty of Medicine Imperial College London London UK; ^3^ Blizard Institute, Barts and The London School of Medicine Queen Mary University of London London UK

**Keywords:** epithelial surface area, glucagon, insulin, L‐cells

## Abstract

**Background and Aims:**

Malnutrition disorders are associated with an enteropathy characterized by villus blunting, secretory cell loss, and mucosal inflammation. To investigate the contribution of undernutrition to enteropathy we conducted a case–control study of adult patients with starvation due to benign esophageal strictures, compared with non‐malnourished community controls.

**Methods:**

Fasting blood samples and duodenal biopsies were collected from 34 cases and 66 controls. Duodenal biopsies were evaluated by morphometry while circulating C‐reactive protein and hormones were measured by ELISA or Luminex. Multivariable models were adjusted for stricture, body mass index, HIV, and C‐reactive protein.

**Results:**

Epithelial surface area was reduced in cases (median 323 μm (IQR 271, 392) per 100 μm muscularis mucosae) compared to controls (448 μm (IQR 384, 516); *p* = 0.0001), even after adjustment in a linear regression model that included BMI, CRP, and HIV status. Hormonal analysis showed lower median concentrations of C‐peptide (0.36 ng/mL vs. 0.7 ng/mL; *p* = 0.003), insulin (639 pg/mL vs. 2037 pg/mL; *p* = 0.0001), and GLP‐2 (0 ng/mL vs. 2.3 ng/mL; *p* = 0.0063) in cases compared to controls. Cases also had higher glucagon (52.4 pg/mL vs. 36.6 pg/mL; *p* = 0.0012) and secretin concentrations (24.4 pg/mL vs. 11.7 pg/mL; *p* = 0.009).

**Conclusion:**

Starvation and malnutrition consequent upon esophageal stricturing drives a further reduction in epithelial surface area, superimposed on environmental enteropathy. There was some evidence of abnormalities in secretion of pancreatic hormones and GLP‐2.

## 1 Introduction

Malnutrition has been a scourge of human populations since time immemorial [1, 2, 3], and it is still a major obstacle to the achievement of the Sustainable Development Goals [4]. Globally, stunting affects millions of children [5], which underscores the importance of the enteropathy that contributes to it. Intuitively, we would expect that the structural and physiological changes consequent to starvation or semi‐starvation should be readily reversible upon restoration of the nutrient supply, but this does not always occur. Stunting, impaired linear growth in young children, is not reliably corrected, at least in childhood, by nutritional supplementation [6], and no more than one‐fifth of severe stunting can be prevented by nutritional interventions, even optimal lipid‐based supplements [7].

Children with Severe Acute Malnutrition (SAM), defined by wasting or nutritional edema, continue to experience far higher mortality rates than would be anticipated if nutritional therapy could easily reverse wasting or edema [8, 9]. Clearly, additional pathophysiological processes contribute to the persistence of malnutrition and its consequent mortality, and it has long been postulated that malnutrition enteropathies compromise digestion and absorption of nutrients, increase permeability and microbial translocation, and drive mucosal and systemic inflammation [10, 11, 12, 13, 14] complicating recovery from a nutritional crisis. Children with SAM or stunting have an enteropathy of varying severity, characterized by villus blunting, inflammation in the lamina propria and epithelium, and loss of secretory cells (goblet cells and Paneth cells) [15, 16, 17, 18]. Nonspecific duodenitis has been reported in children with stunting [19, 20], but no correlations have been shown between anthropometric measures and composite histologic scores [21].

There is evidence of intestinal damage in some, but not all, situations of extreme starvation and in animal models, but only rarely has it been possible to examine the starved human gut microscopically [22]. In most human situations several factors simultaneously contribute to the development of the enteropathy observed, including polymicrobial infections with intestinal pathogens [23, 24, 25] and/or alterations in the microbiota [26]. It is therefore difficult to disaggregate those features of enteropathy due to nutrient deprivation from those due to intestinal pathogens or pathobionts.

In an attempt to quantify mucosal damage due solely to nutrient deprivation, we carried out a study of adult patients with starvation due to caustic esophageal strictures and compared them with controls. These patients, drawn from a population where environmental enteropathy is highly prevalent, presented with dysphagia and malnutrition some weeks after the initial ingestion of caustic substances, when the esophagitis and ulceration had largely healed and the esophageal lesion is a smooth fibrotic stricture, apparently uninflamed. In this situation it was possible to observe the effects of pure undernutrition on the small intestine by assessment of duodenal biopsies.

## 2 Methods

Patients were considered for inclusion once referred to the endoscopy unit of the University Teaching Hospital, Lusaka, for dilatation of benign esophageal strictures. Duodenal biopsies were taken after the endoscopic dilatation, provided there was no additional gastroduodenal pathology (notably pyloric stenosis) which precluded duodenal intubation. Ethical approval was obtained from the University of Zambia Biomedical Research Ethics Committee (006‐11‐15, dated January 12, 2016). Patients were assessed by the endoscopists (V.K., M.M., or P.K.) prior to endoscopy, and written informed consent was obtained. Patients were sedated with midazolam or diazepam, as available, combined with pethidine in doses appropriate for their functional status and intended therapy. Endoscopy was performed using a Pentax 2990i gastroscope using Savary–Gilliard dilators in the majority of instances and occasionally using balloon dilatation. Following successful dilatation, a complete endoscopic examination was performed and up to five biopsies were obtained from the second or third part of the duodenum, orientated and processed.

A second group of non‐malnourished volunteers was recruited from Misisi, the same community where we have previously recruited volunteers for intestinal biopsy studies [27], and where we have carried out studies in children with stunting [23]. These controls were recruited by door‐to‐door recruitment and a three‐stage consent process (as previously described [28]). Endoscopy and biopsy collection was performed as described above. The control participants were recruited as part of the GI Tools study [29] and ethical approval was also obtained from the University of Zambia Biomedical Research Ethics Committee (2291‐2021 dated December 20, 2021).

### 2.1 Biopsy Evaluation

Biopsies from both groups were collected into normal (0.9%) saline and orientated under a dissecting microscope, then placed on cellulose acetate paper (Sartorius GmbH, Gottingen, Germany) in neutral buffered formal saline (CellPath Ltd, Newtown, Powys, UK) and processed into wax blocks. Sections of 3–4 μm were stained with haematoxylin and eosin and then scanned on an Olympus VS120 slide scanning microscope at 20× and 40× magnification, as previously described [16], to generate measurements of villus height (VH), crypt depth (CD), and epithelial perimeter in relation to length of muscularis mucosae. Epithelial perimeter measurements were taken as a proxy measure of brush border surface area. Morphometry was carried out on well‐orientated mucosa (Figure S1).

The criterion for orientation of a section or part of a section was that each crypt should be seen throughout its length. Where this criterion was satisfied, all villus and crypt units, the overlying epithelium, and the underlying muscularis mucosae, were measured. Special care was taken to include small villus units which represent the proximal and distal ends of ridge‐like villi. The clinical and lab staff responsible for collection and processing of the biopsies were the same for both studies. Morphometry was carried out by one observer (C.M.) and confirmed by another (P.K.) to avoid bias.

### 2.2 Anthropometry

Anthropometry for cases and controls included measurement of weight, height, and mid upper arm circumference (MUAC). In patients too weak to stand only MUAC could be measured. We considered MUAC less than 23.5 cm or 20 cm to be indicative of wasting and severe wasting respectively [30].

### 2.3 Blood Testing

Blood samples were available from most, but not all, patients, including some from whom no biopsies could be obtained. Blood samples were collected in the fasted state, either just before or just after endoscopy. No anti‐protease inhibitors were added to the samples during collection, but samples were processed within 30 min of collection and in instances where this was not possible, samples were stored in a 2°C–8°C fridge before processing. HIV testing was done using the Determine HIV‐1/2 test kit (Abbott Diagnostics Medical, Japan). The Milliplex Metabolic Hormone Panel V3 (Merck Millipore, Germany) was used to measure a panel of seven metabolic hormones while C‐reactive protein (Bio‐Techne, USA) and Glucagon‐like peptide–2 (GLP‐2; Merck Millipore, Germany) were measured individually by ELISA. All experiments were run according to the manufacturer's instructions; samples for CRP testing were run at a 1000‐fold dilution while all other assays had samples run neat.

### 2.4 Data Analysis

Clinical and morphometric characteristics were described using median and interquartile range (IQR) as data was found to be not normally distributed using the Shapiro–Wilk test. The primary comparison was mucosal morphometry in those with strictures compared to controls, which was tested using the Kruskal–Wallis test. All other analyzes were considered secondary. Comparisons of circulating hormones and HIV by group were done using Kruskal–Wallis while the Spearman's test was used to assess correlation between BMI and MUAC as well as hormone levels. These endpoints did not change during the course of the study or during post hoc analysis. In all cases, a *p*‐value of less than 0.05 was considered statistically significant. Fractional polynomial regression was employed to model the nonlinear relationship between ghrelin and BMI. A second‐degree polynomial model was fitted where *Y* is ghrelin and *X* is BMI. Although the Shapiro–Wilk test confirmed that epithelial surface area was non‐normally distributed, the departure from normality was modest as the mean and median were very close, a histogram showed very few outliers, and the interquartile range was close to symmetrical. For these reasons, a multivariable linear regression model was used to adjust for the effect of strictures, BMI, HIV and CRP on ESA. Data analysis was conducted in Stata 17 (Corp College Station TX, USA). Missing data was treated as missing, and no imputation was made.

## 3 Results

Patients with esophageal strictures were recruited and biopsied between December 8, 2016 and May 15, 2023. These patients were severely malnourished overall (Table 1), with the majority having had severely restricted food intake for days or weeks prior to the procedure. Over this period, 42 patients with caustic esophageal strictures were assessed for inclusion. Of the 42 assessed, six (6) were excluded due to inability to dilate enough to permit duodenal intubation, resulting in a total of 36 participants who donated endoscopic biopsies. Two (2) of the 36 patients were unable to provide blood alongside biopsy samples, resulting in a total of 34 patients that were included in the final analysis. Controls were recruited and biopsied between August 31, 2022 and July 6, 2023. Of the 80 controls recruited, five (5) did not attend for the procedure on the appointed day, and failure to sedate one individual resulted in 74 endoscopies being conducted. We were unable to collect blood and/or tissue samples in eight (8) participants, giving us a total of 66 included in the final analysis (Figure S2). Karnofsky performance scores clearly show the substantial differences between the patients with strictures and controls (Table 1). Analysis of tissue or blood samples was dependent on the availability of samples and reagents and only a subset of control samples had hormone results. A total of 14 stricture and 38 control participants had results for both morphometry and blood (Figure S3).

**Table 1:** Clinical and demographic characteristics of participants.

	Cases	Controls	*p*
Number	34	66	
Sex (M:F)	19:15	27:39	0.16
Age (median, IQR)	29 (22, 44); *n* = 33	32 (25, 43); *n* = 66	0.48
Body mass index (kg/m^2^)	17.5 (15.6, 19)	23.3 (20.7, 26.7)	0.0001
*n*, BMI range	*n* = 30 (12.9–27.7)	*n* = 66 (17.3–39.7)	
Mid upper arm circumference (cm), *n*	22.9 (20.3, 24.5), *n* = 24	28 (26.2, 31.5), *n* = 66	0.0001
HIV seropositive n (%)	18/32 (56%)	19/66 (29%)	0.01
Educational attainment:			
None	0	7	0.001
Primary	4	22	
Secondary	15	37	
tertiary	9	0	
	(*n* = 28)	(*n* = 66)	
Salaried or full‐time occupation?	17/31 (55%)	44/59 (75%)	0.06
Cigarette smoker currently (%)	3/31 (10%)	14/66 (21%)	0.17
Takes alcohol currently (%)	29/34 (86%)	48/66 (73%)	0.16
Karnofsky score (*n*)	60 (30, 80)	100 (100, 100)	0.001
	[all scores 80 or less] *n* = 22	[all scores 90 or 100] *n* = 63	
C‐reactive protein (mg/L)	4.5 (0.9, 19.2)	2.1 (1.2, 6.2)	0.22
Proportion with high CRP (> 10 mg/L)	9/25 (36%)	7/42 (17%)	

### 3.1 Anthropometry

Many of the stricture patients were weak, with low Karnofsky scores, and unable to stand for height measurements, so full anthropometry measurements are not available for all patients. The majority of stricture patients (Table 1) were undernourished with BMI < 18.5 kg/m^2^ and/or MUAC < 23.5 cm. Eight (8) of the stricture patients had severe wasting, BMI < 16 kg/m^2^ and/or MUAC < 20 cm. Consequently, BMI was lower in cases and strongly correlated with MUAC in those in whom both measurements could be made (Figure S4; *ρ* = 0.89; *n* = 89; *p* < 0.001). One patient had nutritional edema at the time of endoscopy.

### 3.2 Morphometry

**Figure 1:**
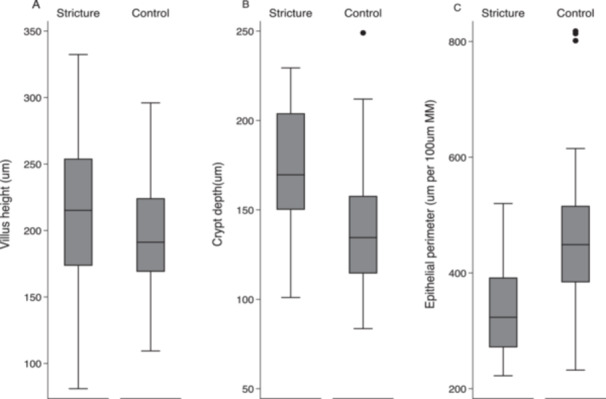
Morphometric measures in stricture patients and controls. Hypothesis testing was performed using the Kruskal–Wallis test: (A) Villus Height (215 μm (IQR 174, 254) Stricture; 191 μm (IQR 169, 224) Controls; *p* = 0.25), (B) Crypt Depth (170 μm (IQR 150, 204) Stricture; 135 μm (IQR 114, 158) Controls; *p* = 0.001), (C) Epithelial Surface Area (323 μm (IQR 271, 392) Stricture; 449 μm (IQR 384, 516) Controls; *p* < 0.001).

Morphometric analysis was performed in well‐orientated biopsies from 23/34 stricture patients and 61/66 controls. Morphometric assessment revealed significantly increased crypt depth in stricture patients, and significantly reduced epithelial perimeter (Table 2; Figure 1). Crypt depth was also increased in HIV seropositive (162.6 μm; IQR 133.6, 182.8) compared to seronegative (133.4 μm; IQR 114.5, 166.1; *p* = 0.033) individuals (Figure S5). Villus height did not differ in cases and controls.

**Table 2:** Mucosal morphometry in biopsies and circulating blood hormone levels from patients with esophageal strictures and healthy controls in Lusaka.

Parameter	Stricture patients (*n* = 34)	*n*	Controls (*n* = 66)	*n*	*p*	Reference range for fasted state
Villus height (μm)	215 (174, 254)	23	191 (169, 224)	61	0.25	356.85 +/− 19.45 (males) [31]
						415.45 +/− 149.8 (females)
Crypt depth (μm)	170 (150, 204)	23	135 (114, 158)	61	0.001	141.3 +/− 6.98 (males)[31]
						176.13 +/− 9.4 (females)
Epithelial perimeter (μm per 100 μm muscularis mucosae)	323 (271, 392)	23	449 (384, 516)	61	0.0001	532.08 +/− 113.85 (males)[31]
						517.37 +/− 160.7 (females)
Insulin (pg/mL)	639.3 (503.7, 993.40)	22	2037 (1429, 2461)	41	0.0001	90.6–867.7 pg/mL [32]
C‐peptide (ng/mL)	0.36 (0.2, 0.5)	25	0.7 (0.4, 0.9)	41	0.0003	0.9–4.3 ng/mL [32]
Glucagon (pg/mL)	52.4 (45.6, 62.7)	22	36.6 (28.5, 47.3)	41	0.001	75–95 pg/mL [33]
GLP‐1 (pg/mL)	37.8 (32.4, 50.3)	21	30.5(24.8, 38.6)	40	0.06	9.3 pg/mL (3.0–33.5; 2.5–97.5th percentile) [34]
						50–66 pg/mL [35]
GLP‐2 (ng/mL)	0 (0, 3.0)	23	2.3 (1.1, 3.3)	31	0.006	3.41 +/− 1.37 ng/mL [36]
						2.29 +/− 1.06 ng/mL [37]
PYY (pg/mL)	152.5 (141.1, 191.8)	21	163.9 (150.3, 176.8)	41	0.30	60.42 +/− 31.48 pg/mL [36]
						61.85 +/− 33.99 pg/mL [37]
Ghrelin (pg/mL)	122.6 (94.4, 159.8)	21	105.2 (91.6, 118.7)	41	0.17	449.40 +/− 203.11 pg/mL [36]
						796.08 +/− 266.79 pg/mL [37]
Secretin (pg/mL)	24.4 (18.7, 38.5)	11	11.7 (8.4, 17.6)	40	0.005	4.4 +/− 0.38 pg/mL [38]
						6.7 +/− 0.5 pg/mL [39]

*Note:* All data expressed as median and interquartile range (IQR).

Abbreviations: GLP‐1, glucagon‐like peptide–1; GLP‐2, glucagon‐like peptide–2; PYY, peptide tyrosine tyrosine.

*p* values refer to results of Kruskal–Wallis test.

### 3.3 Circulating Gut Hormone Concentrations

**Figure 2:**
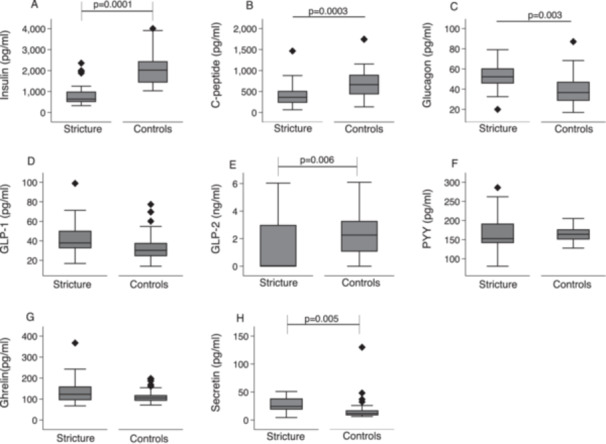
Fasting concentrations of gut hormones in stricture patients and controls. Kruskal–Wallis testing showed significant differences in (A) Insulin (639.3 pg/mL (IQR 503.7,993.40) Stricture; 2037 pg/mL (IQR 1429,2461) Controls; *p* < 0.001), (B) C‐peptide (0.36 pg/mL (IQR 0.2, 0.5) Stricture; 0.7 pg/mL (IQR 0.4, 0.9) Controls; *p* < 0.001), (C) Glucagon (52.4 pg/mL (IQR 45.6, 62.7) Stricture; 36.6 pg/mL (IQR 28.5,47.3) Controls; *p* = 0.003), (E) GLP‐2 (0 pg/mL (IQR 0, 3.0) Stricture; 2.3 pg/mL (IQR 1.1,3.3) Controls; *p* = 0.006) and (H) Secretin (24.4 pg/mL (IQR 18.7, 38.5) Stricture; 11.7 pg/mL (8.4, 17.6) Controls; *p* = 0.005).

Circulating gut hormone concentrations were measured in 25 cases and 43 controls. Stricture patients had reduced levels of insulin, C‐peptide and GLP‐2 compared to controls while glucagon and secretin were increased (Table 2; Figure 2). Sixteen (70%) of 23 stricture patients had undetectable GLP‐2 levels.

**Figure 3:**
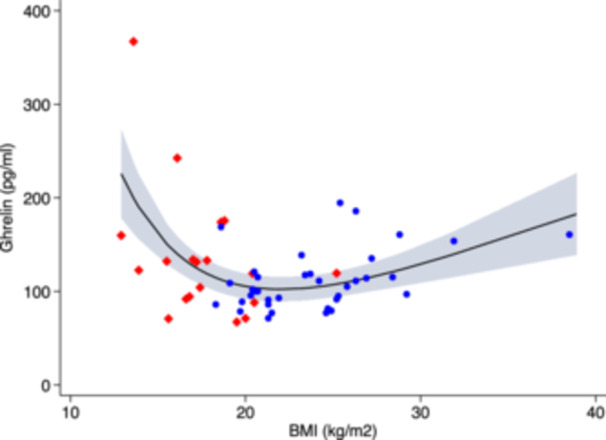
Ghrelin levels in relation to body mass index (BMI) in stricture patients (red diamonds) and controls (blue circles). The curve fit was generated using fractional polynomial regression (*R* ^2^ = 0.29, AIC 630.48), with 95% confidence limits shown in gray.

No significant differences were noted in the levels of PYY and ghrelin, while GLP‐1 showed a borderline difference between cases (37.8 pg/mL (IQR 32.4, 50.3) and controls; 30.5 pg/mL (IQR 24.8, 38.6); *p* = 0.060). A second‐degree fractional polynomial model provided the best fit (*R* ^2^ = 0.29, AIC 630.48) with the coefficients indicating a curvilinear association between fasted ghrelin and BMI (β_1_ = 28,890.6, *p* < 0.001; β_2_ = −12,310; *p* < 0.001). Ghrelin levels were elevated in those individuals who did not fall within the normal BMI range, with the highest levels noted in those who were underweight (Figure 3).

When compared with reported reference values, ghrelin levels were lower overall in our population, while PYY and GLP‐1 were elevated. In the stricture patients, GLP‐1 (*ρ* = 0.50; *n* = 21; *p* = 0.019) and C‐peptide (*ρ* = 0.41; *n* = 25; *p* = 0.043) correlated with CRP, but when analyzed together with controls, only the correlation with GLP‐1 held constant (*ρ* = 0.31; *n* = 60; *p* = 0.017).

**Figure 4:**
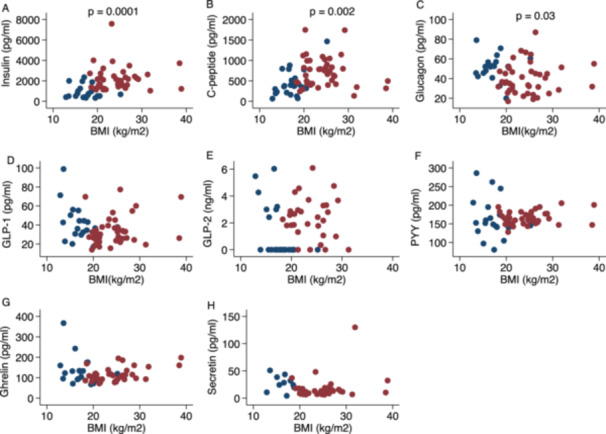
Scatter plots of the different hormones against BMI. Correlations with Insulin (*ρ* = 0.48; *n* = 62; *p* < 0.001), C‐peptide (*ρ* = 0.39; *n* = 65; *p* = 0.002) and glucagon (*ρ* = −0.28; *n* = 60; *p* = 0.03; Figure 4A–C) were noted. Blue circles represent stricture cases while controls are in maroon.

Analysis of fasting hormone levels by BMI revealed correlations with C‐peptide (*ρ* = 0.39; *n* = 65; *p* = 0.002), insulin (*ρ* = 0.47; *n* = 62; *p* < 0.001) and glucagon (*ρ *= −0.28; *n* = 60; *p* = 0.03) as shown in Table 2 and Figure 4. Other pairwise correlations were not significant.

### 3.4 Effect of HIV Infection

There were more HIV seropositive stricture patients (18/32; 56%) than controls (19/66; 29%) (OR 0.32, 95%CI (0.14, 0.79)) and the HIV seropositive participants had higher CRP (7.26 mg/mL HIV positive; 1.50 mg/mL HIV negative; *p* < 0.001) and secretin (18.68 pg/mL HIV positive; 10.72 pg/mL HIV negative; *p* = 0.03) levels (Figure S6).

### 3.5 Multivariable Regression Models

In a multivariable linear regression model, we adjusted the effect of stricture on ESA for BMI, HIV and CRP and found that ESA was still lower in patients with strictures after adjustment. BMI and CRP were not significantly associated. In the final model that confirmed that ESA was lower in patients with strictures, ESA was lower by 93.7 µm (95% CI: 34.1, 153.2; *p* < 0.001) in those with strictures and lower by 60 µm (95% CI: 114.3, 6.7; *p* = 0.03) in those with HIV.

## 4 Discussion

In the belief that adults with severe wasting due to fibrotic esophageal strictures represent a group with “pure” undernutrition, with only modest contributions from inflammation or infection, we studied duodenal mucosal morphology in patients undergoing endoscopic stricture dilatation. These patients presented with strictures months after caustic ingestion and although we cannot completely exclude duodenal injury resulting from caustic ingestion, there was no evidence of macroscopic damage. Given the rapid turnover of duodenal epithelial cells (3–5 days), we assumed that any initial injury would have resolved by the time of the procedure. Nonetheless, the extent to which caustic injury alters epithelial regenerative capacity is unclear. Compared to controls, the cases had significantly reduced epithelial surface area and increased crypt depth. Cases were severely malnourished, with very low BMI and/or MUAC measurements, and they had all been starved or semi‐starved for weeks prior to undergoing endoscopic therapy. The observed reduction in epithelial surface area suggests that starvation imposes an additional severity on the underlying environmental and infectious enteropathy which is ubiquitous in vulnerable populations and is believed to contribute to perpetuation and non‐responsiveness of malnutrition disorders. This additional severity is referred to as malnutrition enteropathy, a term we have also applied to the extremely severe mucosal lesions seen in children with Severe Acute Malnutrition [15, 16].

Previous studies in patients who had undergone varying periods of starvation (several days) showed evidence of an absorptive defect of up to 40%, as assessed by the peak xylose concentration in blood after xylose testing [40]. Similar experiments on the effects of fasting on absorptive function by a different group suggested a reduction of up to 47% in mannitol uptake, and no change in permeability [41]. These observations are consistent with reduced surface area due to starvation or semi‐starvation and justify the use of the term malnutrition enteropathy.

The increased crypt depth in patients with starvation is of interest. Increased crypt depth is one of the cardinal histological features of celiac disease, a hyper‐proliferative enteropathy in which epithelial and lamina propria inflammation are prominent. Increased crypt depth is also seen in environmental enteropathy [27], but one study using single cell transcriptomic analysis suggested that environmental enteropathy is characterized by reduced epithelial cell proliferation [42]. Mucosal inflammation has been clearly demonstrated in environmental enteropathy using histology [43], immunostaining [44], and transcriptomic analysis [45]. The dominant Th17 response in EE [45] may support the hypothesis that this inflammation is driven by polymicrobial infection [15], and this may explain the increased crypt depth observed.

We obtained enough blood in many cases to analyze circulating pancreatic and gastrointestinal hormones. We found that stricture patients had increased glucagon levels while C‐peptide and insulin were reduced. The decrease in C‐peptide, which is a by‐product of insulin production is as expected given the decrease in insulin. As glucagon acts as a counter‐regulatory hormone to insulin, the increase in glucagon and concomitant decrease in insulin is consistent with the stress response to trauma [46]. Together with the modestly elevated CRP, these data challenge our assumption that these patients had “pure” undernutrition.

Enteroendocrine cells in the small and large intestine synthesize and release glucagon‐like peptides in response to nutrient ingestion. While GLP‐1 enhances glucose‐stimulated insulin secretion, GLP‐2 stimulates cell proliferation and inhibits apoptosis [47]. The observed reduction in GLP‐2 levels in the cases is consistent with our previous findings of decreased circulating GLP‐2 as stunting progresses in children [48]. This reduction may be due to l‐cell loss or reduced functionality in these malnourished individuals, resulting in a consequent reduction in GLP‐2 production. It may also be a consequence of prolonged starvation or semi‐starvation as GLP‐2 is known to be nutrient‐stimulated [47, 49]. An increase in GLP‐2 levels in the cases would be desirable as it drives intestinal repair and has been used therapeutically to increase intestinal absorptive function in patients with intestinal failure [50, 51, 52]. Although GLP‐1 and GLP‐2 are both translational products of the proglucagon gene and are expected to be secreted in equimolar quantities [47], the shift observed in the molar ratio in blood could be an adaptive response which prioritizes increased GLP‐1 production by EEC L‐cells. It has been postulated that increases in GLP‐1 in malnourished children may serve an adaptive role by reducing appetite [53]. Unfortunately, were unable to assess the levels of appetite in our study participants and further work may be required to definitively show this. Changes in the ratio of secretion of these two hormones have been observed following gastric bypass, in IBD, or in response to GLP‐1 agonist therapy. There is also plasticity in enteroendocrine cell phenotype [49], but the extent to which this drives the observed changes in malnutrition is as yet unexplored. Ghrelin, which is responsible for stimulating appetite and increasing food intake was expected to be significantly different between groups due to the presence of esophageal strictures. However, a U‐shaped relationship was observed with BMI, such that those on both ends of the BMI range had elevated ghrelin. Secretin concentrations were increased in starved patients, which to our knowledge is the first time this has been reported. Further work is required to understand the link, if any, between elevated secretin, trauma and malnutrition enteropathy as secretin is primarily responsible for pancreatic and biliary secretion [54].

Unsurprisingly, HIV seropositive individuals had higher levels of CRP, which is consistent with the inflammatory state associated with the disease [55]. There was also increased crypt depth and hypersecretinemia in these HIV seropositive individuals. The former is entirely consistent with our previous work [56] though the increased secretin has not previously, to our knowledge, been reported.

This study has obvious limitations. No protease inhibitors were used during blood sample collection, and this may have affected the stability of hormones which are rapidly degraded such as GLP‐1, GLP‐2, and PYY. We attempted to minimize the degradation of these hormones by processing the samples immediately after collection, usually within a 30‐min time frame although this was not always possible. In addition, the potential effect of sedation on the gut hormone levels was not considered. As biopsies were collected at the end of a semi‐urgent therapeutic procedure intended to save life, only a limited sample set could be obtained, and no stool samples were collected. In at least six cases, and possibly more as some failed therapeutic procedures might have gone unrecorded, gastric outlet obstruction precluded taking any biopsies at all. As these were often the most severely wasted patients these biopsies would have been of interest, but once surgical therapy was required it was difficult to trace these patients in the hospital wards. Furthermore, it was intended to re‐biopsy these patients after nutritional recovery, but this also proved difficult logistically as our patients generally did not return to clinic after therapy. Undoubtedly, some died. This may explain why there was no significant differences in CRP between the cases and controls. In addition, information on how long the cases had been living with strictures was not available and we were unable to determine the duration of nutrient deprivation, which is usually slowly progressive. The strength of the study lies in the consistency in the way the measurements were made, with almost all biopsies taken and orientated by the same observer (P.K.), and morphometric measurements were made by the same observer (C.M.). All hormone assays were conducted in one batch. We also know that the undernutrition in our patients was severe and often extreme, so that our hypothesis was given a good test. Our inability to measure fasting glucose in these samples limits our conclusions regarding glucagon and ghrelin levels.

4.1

So, if these conclusions can be generalized, what would be the implications for adults or children with malnutrition? Current thinking is that the combination of nutrient deprivation and polymicrobial infection underlies environmental enteropathy. Our data suggest that severe malnutrition exacerbates the severity of enteropathy, with a reduction of epithelial surface area. This would be predicted to reduce the efficiency of digestion by brush border enzymes, many of which (such as sucrase‐isomaltase, lactase, maltase‐glucoamylase, peptidases and enterokinase) are located in the brush border. Enterokinase is important for activating some other key enzymes, such as trypsinogen, and the brush border is also the location of a huge array of solute transporters required for uptake of multiple nutrients. Reduced brush border surface area would explain many of the gene expression deficits we have previously reported [56, 57]. If indeed malnutrition enteropathy does prolong malnutrition and reduce response to therapy, therapies directed at mucosal healing could be of great benefit in attempting to reduce mortality in adults and children with severe malnutrition.

## Author Contributions

**Ellen Besa:** methodology, software, data curation, formal analysis, investigation, validation, visualization, writing – original draft. **Violet Kayamba:** conceptualization, methodology, data curation, investigation, visualization, validation, formal analysis, project administration, writing – review and editing. **Victor Mudenda:** conceptualization, methodology, data curation, validation, formal analysis, visualization, writing – review and editing. **Chola Mulenga:** methodology, data curation, validation, writing – review and editing, software. **Enala Musheba:** methodology, data curation, software, investigation, validation, writing – review and editing. **Malambo Mubbunu:** methodology, data curation, investigation, writing – review and editing. **Gary Frost:** investigation, validation, supervision, visualization, writing – review and editing. **Paul Kelly:** conceptualization, methodology, software, data curation, investigation, validation, formal analysis, supervision, funding acquisition, visualization, project administration, resources, writing – original draft.

## Conflicts of Interest

The authors declare no conflicts of interest.

## Supporting information


Supporting File 1

Supporting File 2

Supporting File 3

Supporting File 4

Supporting File 5

Supporting File 6

## Data Availability

The data described in the manuscript, code book and analytic code will be made available on request from the corresponding author. The data are not publicly available due to privacy or ethical restrictions.
