# Supplementary figures and images for: Structural and Hormonal Changes Associated With Starvation in Zambian Adult Patients With Esophageal Strictures: A Cross‐Sectional Study
Source: Health Sci Rep. 2026 Jul 11;9(7):e72772. doi: 10.1002/hsr2.72772 (PMC13355291; doi:10.1002/hsr2.72772)

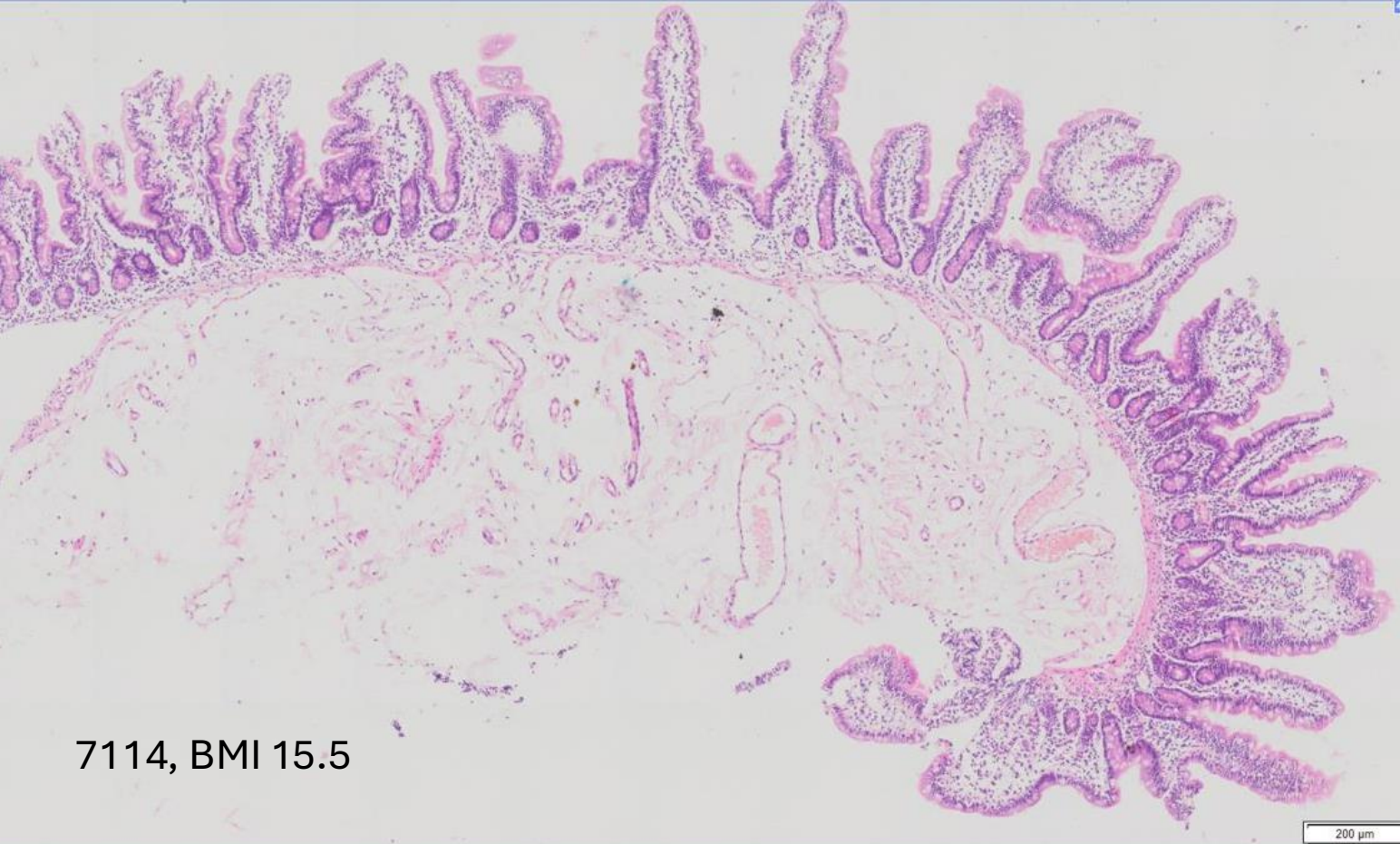

7114, BMI 15.5

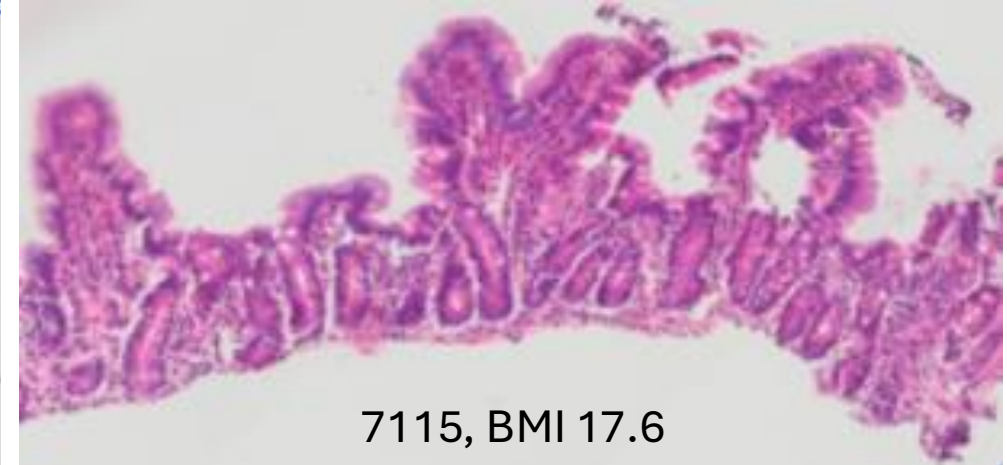

7115, BMI 17.6

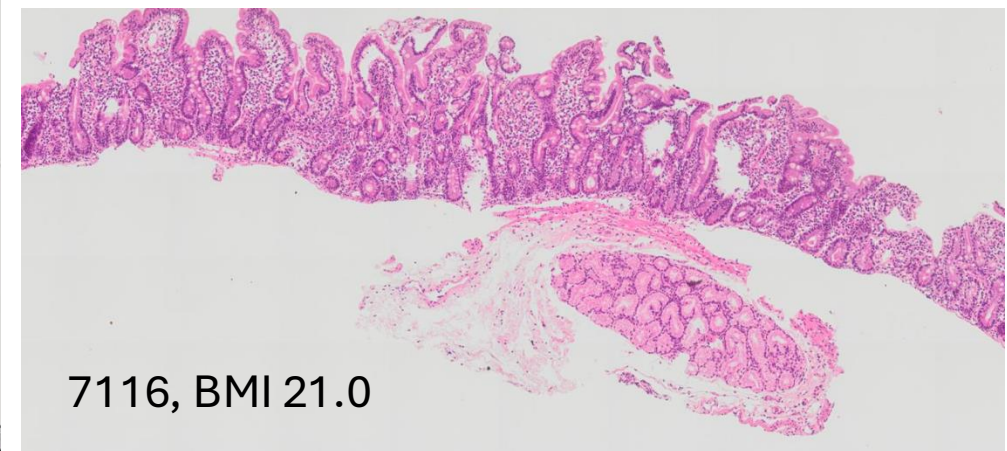

7116, BMI 21.0

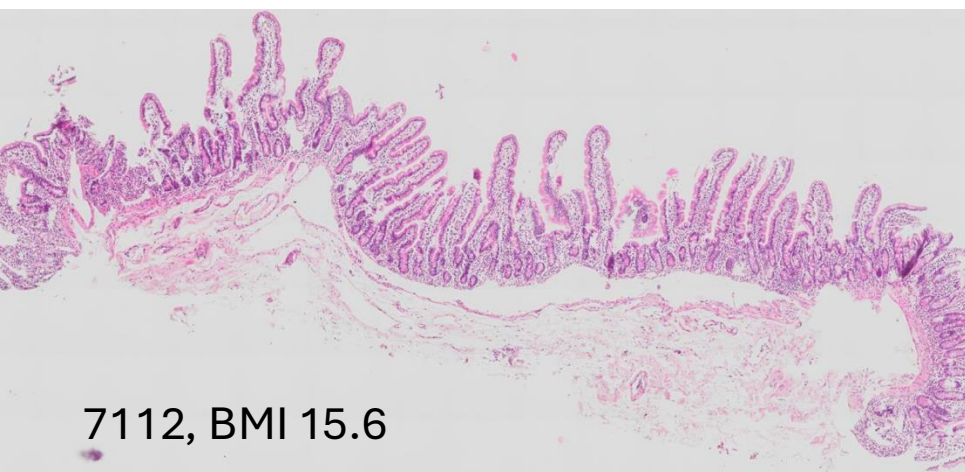

7112, BMI 15.6

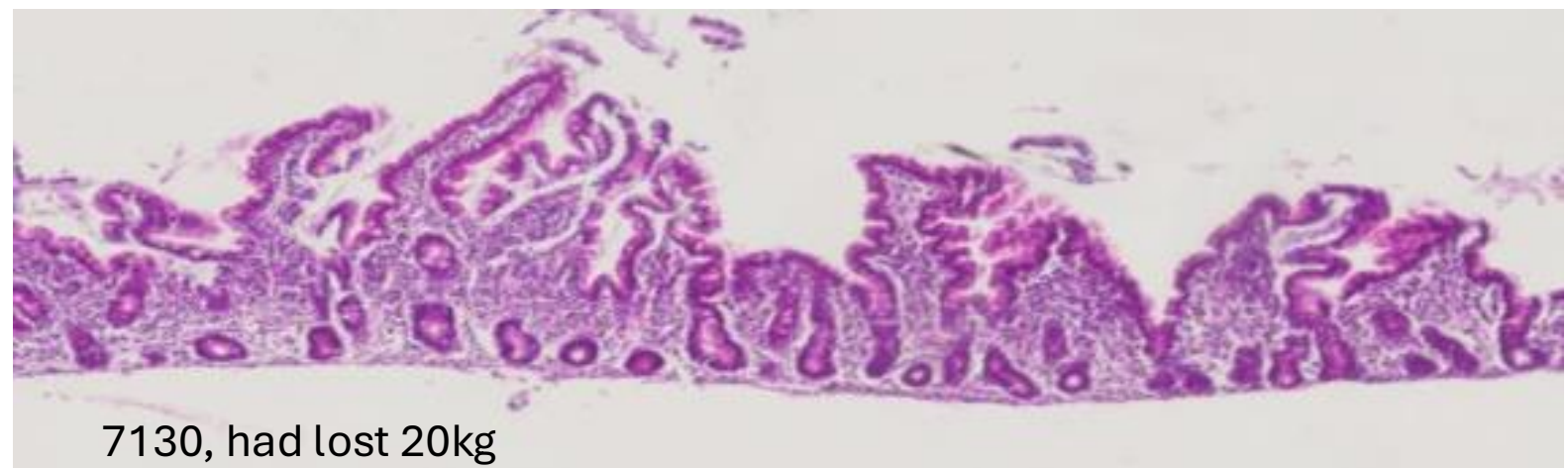

7130, had lost 20kg

Supplement: Supplementary file 1 — Supporting File 1 [file HSR2-9-e72772-s003.pdf]
