# Supplementary material for: Structural and Hormonal Changes Associated With Starvation in Zambian Adult Patients With Esophageal Strictures: A Cross‐Sectional Study
Source: Health Sci Rep. 2026 Jul 11;9(7):e72772. doi: 10.1002/hsr2.72772 (PMC13355291; doi:10.1002/hsr2.72772)

MALNUTRITION ENTEROPATHY: STRUCTURAL AND HORMONAL CHANGES ASSOCIATED WITH STARVATION IN ZAMBIAN PATIENTS WITH OESOPHAGEAL STRICTURES

Besa Ellen

Supplementary Figure 2: Flow chart of participant recruitment

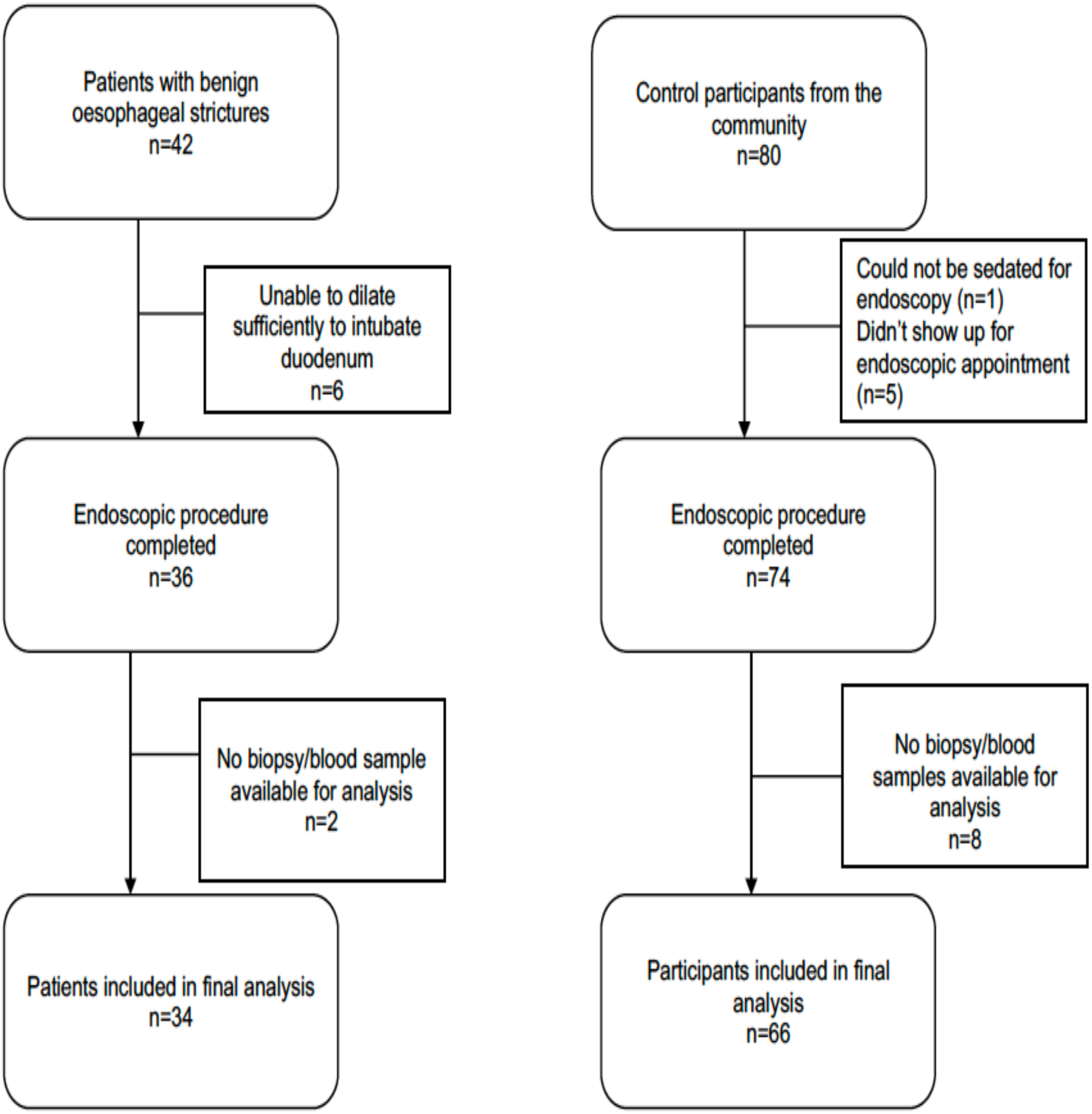

Supplement: Supplementary file 2 — Supporting File 2 [file HSR2-9-e72772-s001.pdf]
