# Supplementary material for: Structural and Hormonal Changes Associated With Starvation in Zambian Adult Patients With Esophageal Strictures: A Cross‐Sectional Study
Source: Health Sci Rep. 2026 Jul 11;9(7):e72772. doi: 10.1002/hsr2.72772 (PMC13355291; doi:10.1002/hsr2.72772)

MALNUTRITION ENTEROPATHY: STRUCTURAL AND HORMONAL CHANGES ASSOCIATED WITH STARVATION IN ZAMBIAN PATIENTS WITH OESOPHAGEAL STRICTURES

Besa Ellen

Supplementary Figure S4: Correlation between body mass index (BMI) and mid upper arm circumference (MUAC; ρ=0.90; n=89; p<.001).

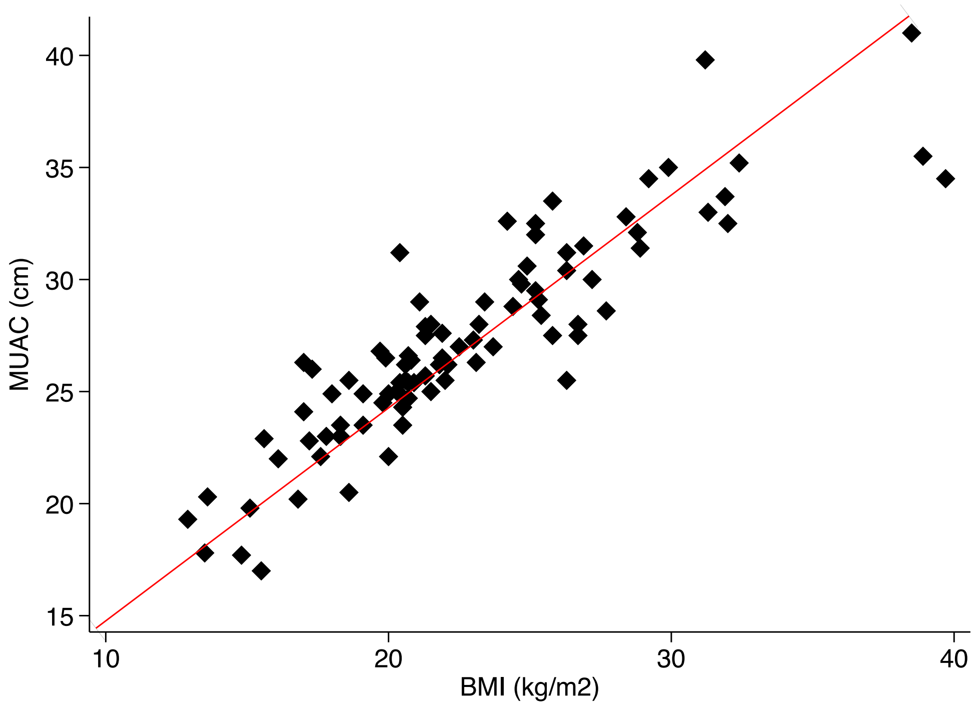

Supplement: Supplementary file 4 — Supporting File 4 [file HSR2-9-e72772-s005.pdf]
