# Supplementary material for: Structural and Hormonal Changes Associated With Starvation in Zambian Adult Patients With Esophageal Strictures: A Cross‐Sectional Study
Source: Health Sci Rep. 2026 Jul 11;9(7):e72772. doi: 10.1002/hsr2.72772 (PMC13355291; doi:10.1002/hsr2.72772)

MALNUTRITION ENTEROPATHY: STRUCTURAL AND HORMONAL CHANGES ASSOCIATED WITH STARVATION IN ZAMBIAN PATIENTS WITH OESOPHAGEAL STRICTURES

Besa Ellen

Supplementary Figure S5: Analysis by HIV status showed significant differences in CD (HIV Seronegative; 133.4μm (IQR 114.5, 166.1); HIV seropositive; 162.6μm (IQR 133.6, 182.8); p=0.033)

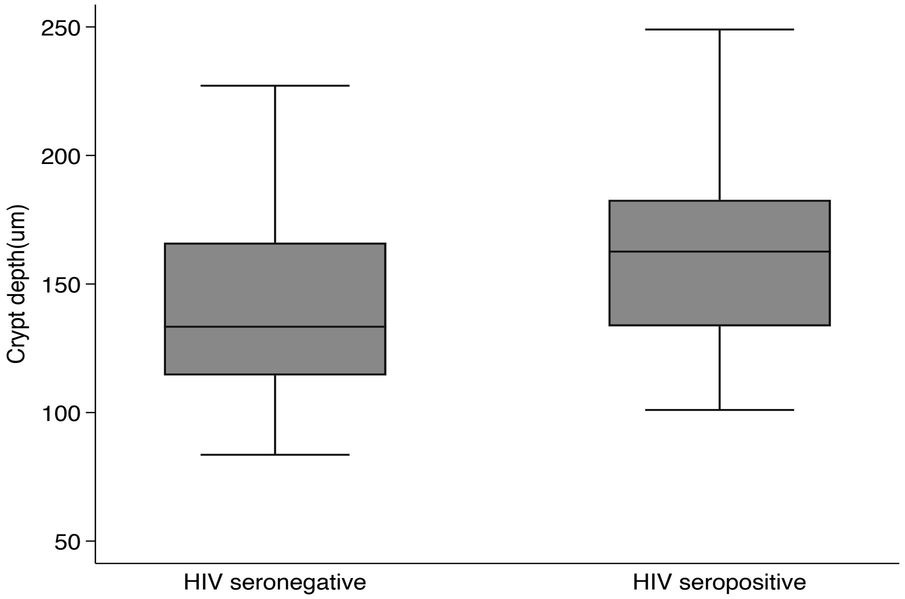

Supplement: Supplementary file 5 — Supporting File 5 [file HSR2-9-e72772-s002.pdf]
