# Supplementary material for: Structural and Hormonal Changes Associated With Starvation in Zambian Adult Patients With Esophageal Strictures: A Cross‐Sectional Study
Source: Health Sci Rep. 2026 Jul 11;9(7):e72772. doi: 10.1002/hsr2.72772 (PMC13355291; doi:10.1002/hsr2.72772)

MALNUTRITION ENTEROPATHY: STRUCTURAL AND HORMONAL CHANGES ASSOCIATED WITH STARVATION IN ZAMBIAN PATIENTS WITH OESOPHAGEAL STRICTURES

Besa Ellen

Supplementary Figure S6: Analysis by HIV status showed significant differences in CRP (HIV seropositive 7.26mg/ml; HIV seronegative 1.50mg/ml; p=0.005) and secretin (HIV seropositive 18.68 pg/ml; HIV seronegative 10.72pg/ml; p=0.02) using the Kruskal Wallis test.

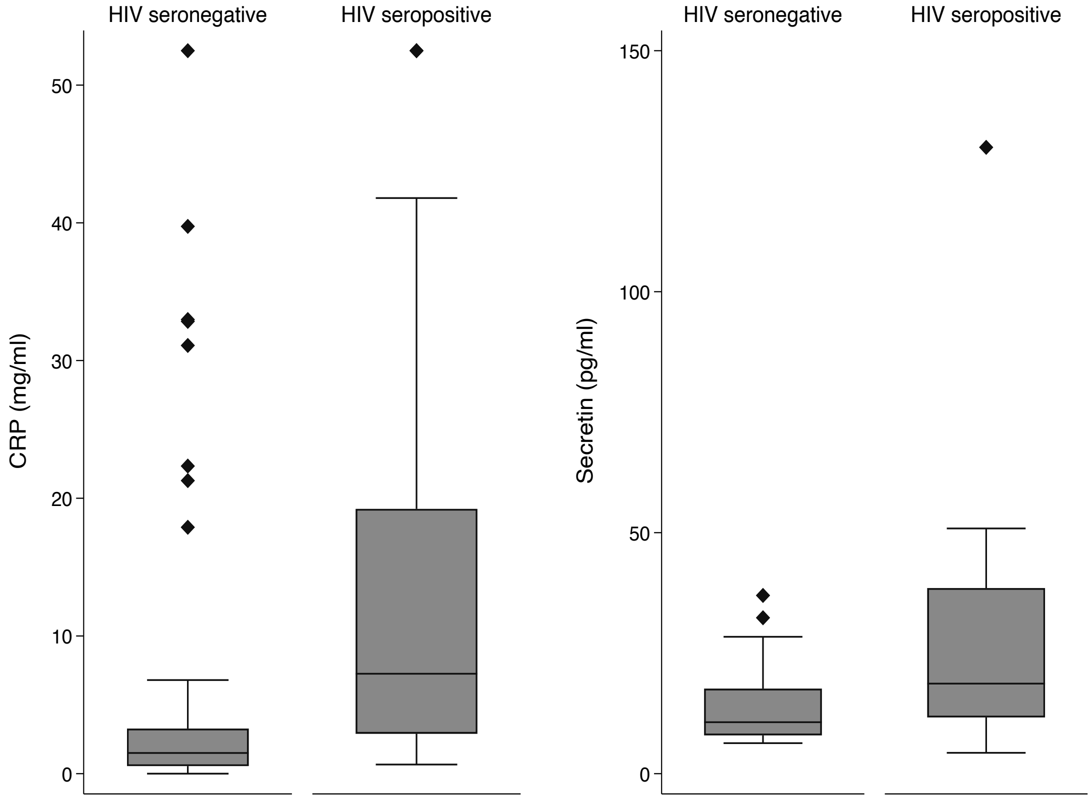

Supplement: Supplementary file 6 — Supporting File 6 [file HSR2-9-e72772-s006.pdf]
